# Adjuvant donafenib plus sintilimab therapy for hepatocellular carcinoma at high risk of recurrence after resection: a retrospective cohort study

**DOI:** 10.3389/fimmu.2026.1736128

**Published:** 2026-10-14

**Authors:** Chenggang Li, Hanchuan Shen, Yang Liu, Tianci Zhao, Chaoxian Li

**Affiliations:** Faculty of Hepato‐Pancreato‐Biliary Surgery, The First Medical Center, Chinese People's Liberation Army (PLA) General Hospital, Beijing, China

**Keywords:** adjuvant therapy, donafenib, hepatocellular carcinoma, PD-1 antibody, propensity score matching, recurrence-free survival, retrospective cohort

## Abstract

**Background:**

Hepatocellular carcinoma (HCC) carries a high recurrence risk after curative resection, yet effective adjuvant strategies remain lacking. Although donafenib plus anti-PD-1 monoclonal antibodies has shown efficacy in advanced HCC, the effectiveness in patients at high recurrence risk after curative resection remains unclear.

**Methods:**

This retrospective cohort study included 143 patients with high-risk HCC after curative resection between June 2019 and April 2024. Patients received adjuvant donafenib plus sintilimab (n=76) or active surveillance (n=67). IPTW, stabilized IPTW (sIPTW), and propensity score matching (PSM) were applied to reduce baseline imbalance. The primary endpoint was recurrence-free survival (RFS), with overall survival (OS) and treatment-emergent adverse events (TEAEs) as secondary endpoints. Survival outcomes were assessed using Kaplan–Meier analysis and Cox regression models.

**Results:**

After IPTW adjustment, adjuvant donafenib plus sintilimab was associated with improved RFS compared with surveillance (median RFS, 22.4 vs. 17.4 months; P = 0.036). The 12- and 24-month RFS rates were 78.2% and 44.4% versus 64.4% and 33.2%, respectively. Multivariable Cox analysis confirmed an independent association between adjuvant therapy and reduced recurrence risk (HR, 0.58; 95% CI, 0.35–0.96; P = 0.036), with consistent results in sIPTW and PSM analyses. OS showed a favorable but nonsignificant trend. Subgroup analyses demonstrated a consistent trend toward RFS benefit across most clinically relevant strata. Among treated patients, TEAEs occurred in 72.4%, mainly grade 1–2 events; grade ≥3 events occurred in 7.9%, with no treatment-related deaths.

**Conclusions:**

Adjuvant donafenib plus sintilimab was associated with improved RFS in patients with high-risk HCC, with an acceptable safety profile, supporting its potential as a risk-adapted adjuvant strategy that warrants further validation in prospective studies.

## Introduction

Hepatocellular carcinoma (HCC) causes >900,000 new cases and >830,000 deaths annually, a toll projected to rise in coming decades (1). Although hepatectomy offers curative intent, 50–70% of patients relapse within 5 years and about 30% within 2 years (2). Recurrence is therefore classified as early (< 2 years) or late (≥ 2 years). Early events stem mainly from occult intrahepatic metastases and aggressive tumor traits—microvascular invasion, large or multifocal lesions, high α-fetoprotein, poor differentiation—whereas late events reflect *de-novo* carcinogenesis in chronically diseased liver (3). Among these factors, MVI is particularly relevant to early recurrence because it reflects microscopic tumor emboli within the intrahepatic microvasculature and is considered a pathological surrogate of occult intrahepatic dissemination (4). Patients with viral/alcoholic cirrhosis, ongoing hepatic inflammation, microvascular invasion or narrow margins thus comprise a high-risk cohort requiring intensive surveillance and adjuvant strategies (5). Crucially, early recurrence portends markedly worse survival than late recurrence, highlighting the need to identify and mitigate early-phase risk (6). Recent studies have further highlighted the roles of tumor microenvironment remodeling, immune dysregulation, and angiogenic signaling in HCC progression, providing additional biological rationale for combined targeted and immunotherapeutic strategies (7, 8).

Despite curative hepatectomy, there is still no universally accepted adjuvant strategy for HCC. The 2024 European Association for the Study of the Liver (EASL) and the 2025 American Association for the Study of Liver Diseases (AASLD) guidelines both conclude that no regimen can yet be routinely endorsed outside clinical trials (9). Negative results from the phase III STORM study, in which sorafenib failed to prolong recurrence-free survival (RFS) in an unselected population, underscore the need for precise risk stratification before systemic therapy is deployed (10).

In this context, immunotherapy and targeted therapy combinations have generated considerable attention. The IMbrave050 trial tested atezolizumab plus bevacizumab (T+A) in patients with resected or ablated high-risk HCC. While interim analysis demonstrated a significant RFS advantage (HR 0.72) (11), the 35-month update reported at the 2024 ESMO Congress showed diminishing benefit (HR 0.90) with no overall survival (OS) improvement (12), suggesting that the regimen effectively suppresses early recurrence but has limited impact on late, liver-disease–driven relapse. This study validated the principle that systemic immune–antiangiogenic therapy can alter the natural history of high-risk HCC, paving the way for novel adjuvant strategies. The attenuation of RFS benefit over time highlights the remaining unmet need for more durable adjuvant strategies and provides a rationale for evaluating alternative TKI–ICI combinations, such as donafenib plus sintilimab, in patients with resected high-risk HCC.

Donafenib, a deuterated sorafenib analog, became the first TKI to show superiority over sorafenib in a phase II/III trial (13). Importantly, donafenib was also associated with a more favorable tolerability profile than sorafenib, including a significantly lower incidence of drug-related grade ≥3 adverse events. Early-phase studies of donafenib combined with anti-PD-1 monoclonal antibodies, such as sintilimab, have shown promising efficacy and tolerability in unresectable HCC, with signals of synergistic antitumor activity (14). Whether such next-generation TKI–ICI combinations can recapitulate the efficacy achieved in advanced-stage disease when deployed as adjuvant therapy after curative resection remains an unresolved yet testable hypothesis that underpins this study.

Accordingly, we performed this retrospective cohort study to assess whether adjuvant donafenib combined with sintilimab is associated with a decreased risk of postoperative recurrence and maintain an acceptable safety profile in patients with predominantly HBV-related HCC who harbor established high-risk features after curative resection. The results aim to furnish evidence for risk-adapted postoperative management in this vulnerable cohort.

## Materials and methods

### Study design and population

This retrospective cohort study included patients with HCC who harbored high-risk recurrence factors following curative resection at the First Medical Center, Chinese PLA General Hospital, between June 2019 and April 2024. Inclusion criteria were as follows: (1) age ≥18 years; (2) histologically confirmed HCC; (3) curative resection performed according to the Chinese guidelines for liver cancer; (4) presence of at least one high-risk factor for recurrence, including tumor size ≥5 cm, multiple tumors (≥2), macrovascular invasion (portal vein or hepatic vein), microvascular invasion (MVI), or satellite nodules; (5) Child-Pugh class A liver function; (6) Eastern Cooperative Oncology Group performance status (ECOG PS) of 0–1; (7) no prior systemic or locoregional therapy for HCC before resection; and (8) availability of complete follow-up information. Exclusion criteria were: (1) histological diagnosis of fibrolamellar HCC, sarcomatoid HCC, or mixed cholangiocarcinoma; (2) presence of extrahepatic metastasis; (3) co-infection with hepatitis B virus (HBV) and hepatitis C virus (HCV); (4) recurrence or death within 3 months after surgery; (5) receipt of other adjuvant therapies such as transarterial chemoembolization (TACE), hepatic arterial infusion chemotherapy (HAIC), traditional Chinese medicine, or TKI monotherapy; and (6) loss to follow-up. Patients who experienced recurrence or death within 3 months after surgery were excluded, as the study was designed to evaluate adjuvant systemic therapy among patients who had adequately recovered from surgery and were clinically eligible to initiate postoperative treatment. Events occurring during this very early postoperative period may reflect occult residual disease, perioperative complications, or highly aggressive tumor biology, and may arise before sufficient exposure to adjuvant therapy could be achieved. This study adheres to the STROBE guidelines for the reporting of observational studies (15). Ethical approval for this study was obtained from the Ethics committee of Chinese PLA General Hospital and the need for individual patient consent was waived due to the retrospective nature of the study.

### Treatment procedures

Patients in the combination group received adjuvant therapy beginning 4–8 weeks after surgery, once postoperative recovery was achieved and liver function was stable. The treatment regimen consisted of donafenib, initiated at 0.1 g orally twice daily and subsequently titrated to 0.2 g twice daily according to individual tolerability, combined with the anti-PD-1 monoclonal antibody sintilimab, administered intravenously at 200 mg every 3 weeks. The control group comprised patients who did not receive any postoperative adjuvant treatment.

During treatment, if grade ≥3 adverse events or clinically intolerable toxicities occurred, donafenib therapy could be temporarily interrupted or dose-reduced according to clinical judgment. Treatment could be resumed once toxicity improved to grade ≤1 or to a clinically tolerable level. Permanent discontinuation was considered in cases of persistent or recurrent severe toxicity, intolerable adverse events, or disease recurrence. Immune-related adverse events associated with anti-PD-1 monoclonal antibody were managed according to established clinical guidelines, including temporary interruption or discontinuation of immunotherapy when necessary.

Adjuvant therapy was continued until disease recurrence, unacceptable toxicity, or patient decision to discontinue treatment, with a maximum planned treatment duration of 12 months in routine clinical practice. In routine clinical practice, treatment decisions were made based on multidisciplinary team (MDT) discussion and physicians’ clinical judgment. Before treatment initiation, patients were informed about their estimated risk of recurrence, the uncertainty of evidence regarding adjuvant systemic therapy, the potential benefits and possible toxicities of treatment, as well as alternative options such as active surveillance, and treatment was initiated following shared decision-making between physicians and patients.

### Data collection and follow-up

Baseline demographic, clinical, and pathological data were collected from electronic medical records, including age, sex, ECOG PS, Child-Pugh score, Barcelona Clinic Liver Cancer (BCLC) stage, hepatitis B surface antigen (HBsAg) status, serum alpha-fetoprotein (AFP) levels, tumor number, tumor diameter, presence of portal vein tumor thrombus (PVTT), and MVI. These variables were considered in the baseline balance diagnostics and subsequent analyses. Follow-up was conducted according to routine clinical practice and national guideline recommendations, including contrast-enhanced CT or MRI and serum AFP testing every 3 months during the first two years after surgery, every 6 months between years 2 and 5, and annually thereafter.

### Endpoints

The primary endpoint was RFS, defined as the time from surgery to radiologically or histologically confirmed recurrence or death from any cause, and 12- and 24-month RFS rates. Recurrence was primarily determined based on contrast-enhanced imaging findings (CT or MRI) and could be supported by serum AFP elevation and clinical evaluation when appropriate. Secondary endpoints included OS, defined as the time from surgery to death from any cause, 12- and 24-month OS rates, and treatment-related safety.

### Safety assessment and dose modifications

Safety was assessed through the frequency and severity of treatment-emergent adverse events (TEAEs). Safety outcomes were documented and graded based on the Common Terminology Criteria for Adverse Events (CTCAE) version 5.0. Dose modifications and the management of adverse events (AEs) were conducted in accordance with the official drug prescribing information. For study drug–related adverse events of grade 3 or higher, donafenib was temporarily interrupted. If the adverse event resolved to baseline or grade ≤1 within 2 weeks, treatment could be resumed at a reduced or previously tolerated dose, generally 100 mg twice daily or 200 mg every other day. Permanent discontinuation was considered for persistent or recurrent severe toxicity, or for adverse events that did not resolve sufficiently despite treatment interruption.

### Statistical analysis

Descriptive statistics were used to summarize baseline demographic and clinical characteristics. Continuous variables, such as age, were expressed as means with standard deviations or medians with interquartile ranges, while categorical variables were reported as frequencies and percentages. For the survival analysis, patients who did not experience the event of interest (e.g., recurrence or death) during the study period or were lost to follow-up, were censored at the date of their last clinical follow-up or the data cutoff date, whichever occurred first. To minimize selection bias, inverse probability of treatment weighting (IPTW) and stabilized inverse probability of treatment weighting(sIPTW) were applied. Propensity scores were estimated using a multivariable logistic regression model incorporating baseline variables considered clinically relevant to both postoperative treatment allocation and recurrence risk, including age, sex, HBsAg positivity, AFP ≥400 ng/mL, tumor diameter ≥5 cm, tumor number, BCLC stage, and ECOG PS. To reduce the influence of extreme weights, IPTW and sIPTW weights were truncated at the 95th percentile. The sIPTW was used to mitigate artificial inflation of the sample size and to reduce the likelihood of false-positive results. PSM was performed as a sensitivity analysis for the primary endpoint (RFS), using nearest-neighbor matching with a 1:1 ratio and a caliper width of 0.2 of the standard deviation of the logit of the propensity score. Covariate balance between groups before and after IPTW, sIPTW, and PSM was assessed using standardized mean differences (SMDs) for the prespecified baseline variables included in the baseline characteristics assessment, with an SMD <0.1 indicating acceptable balance. Baseline variables not included in the final propensity score model—including Child-Pugh score, PVTT, and MVI—were evaluated in the balance diagnostics but were not forced into the model, in order to avoid model instability, overfitting, and potential collinearity given the modest size of this retrospective cohort. Survival outcomes were estimated using the Kaplan–Meier method, with differences between curves assessed using the log-rank test. Univariable and multivariable Cox proportional hazards models were applied to identify independent prognostic factors. Variables with a P value <0.1 in the univariate analysis were included in the multivariate analysis. The proportional hazards assumption of the Cox regression model was evaluated using Schoenfeld residual tests. To reduce potential immortal time bias related to differences in treatment initiation after surgery, a landmark analysis was performed. A landmark time point of 8 weeks (56 days) after surgery was selected based on routine clinical practice, and only patients who remained recurrence-free at that time were included in the analysis. Survival outcomes after the landmark time point were evaluated using IPTW-weighted Cox proportional hazards models. Safety analyses were descriptive. Treatment duration was summarized using the median and interquartile range, while 12-month treatment completion, dose reduction, temporary interruption, permanent discontinuation, and documented adverse events were reported as frequencies and percentages. The 12-month completion rate was additionally calculated among patients evaluable for treatment completion at the data cutoff.

## Results

### Patient characteristics

A total of 168 patients who underwent curative resection for HCC were initially screened. After excluding 25 patients (4 with low-risk recurrence and 21 who received other adjuvant therapies), 143 patients with high-risk recurrence factors were included in the final analysis. Among them, 76 patients received adjuvant donafenib plus sintilimab therapy, while 67 patients were assigned to the control group (**Figure 1**).

**Figure 1:**
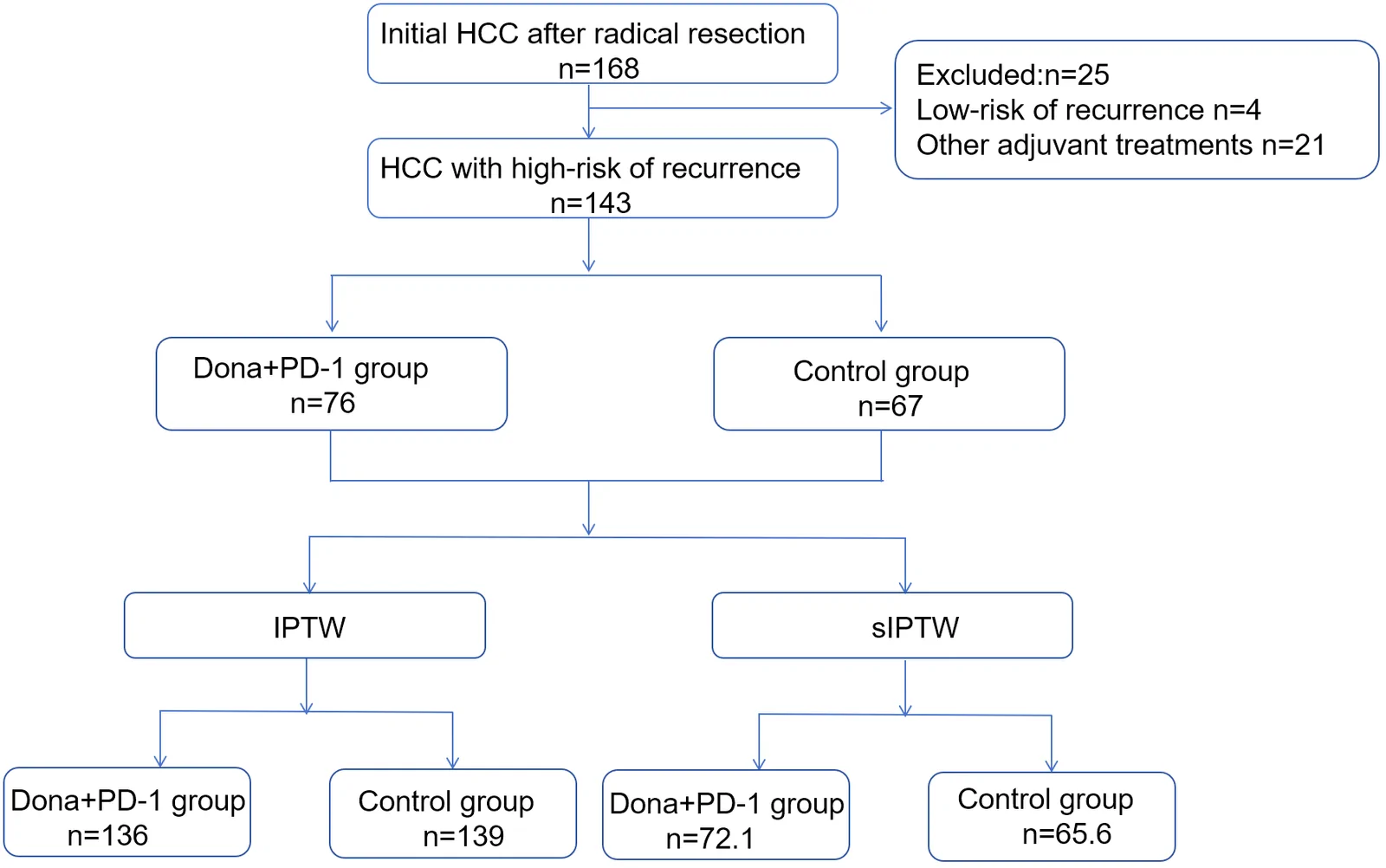
Study flow and propensity-weighting strategy. Flow diagram of the retrospective cohort illustrating screening, exclusion, treatment allocation (donafenib + PD-1 inhibitor vs control), and subsequent application of inverse-probability-of-treatment weighting (IPTW) and stabilized IPTW (sIPTW) to balance baseline covariates; effective sample sizes after weighting are indicated. HCC, hepatocellular carcinoma; PD-1, programmed-death-1; IPTW, inverse-probability-of-treatment weighting.

Before IPTW adjustment, the most notable baseline imbalances involved ECOG performance status, BCLC stage, HBsAg status, and AFP level. ECOG performance status 0 (94.7% vs. 76.1%) and BCLC stage A disease (67.1% vs. 50.7%) were more frequent in the treatment group, whereas BCLC stage C disease (15.8% vs. 37.3%) and HBsAg positivity (72.4% vs. 89.6%) were more frequent in the control group. AFP ≥400 ng/mL was more frequent in the treatment group (39.5% vs. 23.9%). The remaining baseline variables are presented in **Table 1**. After IPTW and sIPTW adjustment, covariate balance was substantially improved, with most variables achieving or approaching the conventional SMD threshold of 0.1, although minor residual imbalance remained for some covariates. The corresponding SMD plots before and after adjustment are shown in **Supplementary Figure S1C**.

**Table 1:** Baseline characteristics of patients before and after IPTW.

Characteristics	Before IPTW				After IPTW				After sIPTW			
	Control Group (n=67)	Treatment group (n=76)	*P*	*SMD*	Control Group (n=139)	Treatment group (n=136)	*P*	*SMD*	Control Group (n=65.6)	Treatment group (n=72.1)	*P*	*SMD*
Age, mean (SD), years	56.99 (12.35)	58.00 (10.18)	0.591	0.090	58.25 (12.28)	58.09 (9.95)	0.939	0.014	58.27 (12.27)	58.07 (9.96)	0.920	0.018
Sex, n (%)			0.950	0.051			0.894	0.023			0.910	0.020
Male	57 (85.1)	66 (86.8)			119.7 (86.0)	118.0 (86.8)			56.5 (86.0)	62.5 (86.7)		
Female	10 (14.9)	10 (13.2)			19.6 (14.0)	18.0 (13.2)			9.2 (14.0)	9.6 (13.3)		
ECOG PS, n (%)			0.003	0.547			0.410	0.157			0.391	0.162
0	51 (76.1)	72 (94.7)			119.7 (86.0)	123.7 (91.0)			56.5 (86.1)	65.7 (91.2)		
1	16 (23.9)	4 ( 5.3)			19.5 (14.0)	12.3 ( 9.0)			9.1 (13.9)	6.3 ( 8.8)		
Child Pugh, n (%)			0.561	0.185			0.644	0.153			0.669	0.153
5	56 (83.6)	65 (85.5)			118.7 (85.2)	115.4 (84.9)			56.0 (85.3)	61.2 (84.8)		
6	11 (16.4)	10 (13.2)			20.5 (14.8)	19.0 (14.0)			9.6 (14.7)	10.1 (14.0)		
7	0 ( 0.0)	1 ( 1.3)			0.0 ( 0.0)	1.6 ( 1.1)			0.0 ( 0.0)	0.8 ( 1.2)		
BCLC, n (%)			0.013	0.503			0.848	0.103			0.845	0.104
A	34 (50.7)	51 (67.1)			82.6 (59.3)	85.6 (63.0)			39.0 (59.4)	45.5 (63.1)		
B	8 (11.9)	13 (17.1)			20.7 (14.9)	21.2 (15.6)			9.8 (14.9)	11.3 (15.6)		
C	25 (37.3)	12 (15.8)			36.0 (25.8)	29.2 (21.4)			16.9 (25.7)	15.3 (21.3)		
HBsAg postive, n (%)			0.018	0.449			0.528	0.117			0.525	0.118
No	7 (10.4)	21 (27.6)			22.1 (15.9)	27.7 (20.4)			10.4 (15.9)	14.7 (20.4)		
Yes	60 (89.6)	55 (72.4)			117.2 (84.1)	108.3 (79.6)			55.2 (84.1)	57.4 (79.6)		
AFP ≥ 400ng/mL, n (%)			0.070	0.340			0.725	0.065			0.749	0.059
No	51 (76.1)	46 (60.5)			95.9 (68.9)	89.5 (65.8)			45.0 (68.5)	47.4 (65.8)		
Yes	16 (23.9)	30 (39.5)			43.3 (31.1)	46.4 (34.2)			20.6 (31.5)	24.7 (34.2)		
Tumor number, n (%)			1.000	0.018			0.967	0.008			0.987	0.003
Single	56 (83.6)	63 (82.9)			113.9 (81.8)	110.9 (81.5)			53.7 (81.8)	58.9 (81.7)		
Multiple	11 (16.4)	13 (17.1)			25.3 (18.2)	25.1 (18.5)			11.9 (18.2)	13.2 (18.3)		
PVTT, n (%)			0.309	0.209			0.675	0.074			0.690	0.071
No	54 (80.6)	67 (88.2)			116.4 (83.6)	117.3 (86.2)			54.9 (83.7)	62.2 (86.2)		
Yes	13 (19.4)	9 (11.8)			22.8 (16.4)	18.7 (13.8)			10.7 (16.3)	9.9 (13.8)		
MVI, n (%)			0.600	0.116			0.839	0.037			0.851	0.034
No	27 (40.3)	35 (46.1)			61.2 (44.0)	62.3 (45.8)			28.9 (44.0)	32.9 (45.7)		
Yes	40 (59.7)	41 (53.9)			78.0 (56.0)	73.7 (54.2)			36.8 (56.0)	39.2 (54.3)		
Tumor diameter ≥ 5cm n (%)			0.383	0.177			0.918	0.018			0.924	0.017
No	24 (35.8)	21 (27.6)			46.1 (33.1)	43.8 (32.2)			21.6 (32.9)	23.1 (32.1)		
Yes	43 (64.2)	55 (72.4)			93.2 (66.9)	92.2 (67.8)			44.1 (67.1)	49.0 (67.9)		

IPTW, Inverse Probability of Treatment Weighting; SD, Standard Deviation; ECOG PS, Eastern Co-operative Oncology Group Performance Status; BCLC, Barcelona Clinic Liver Cancer; HBsAg, Hepatitis B virus; AFP, alpha-fetoprotein; MVI, microvascular invasion.

### Survival analysis

The RFS was significantly longer in the donafenib plus sintilimab group compared with the control group (22.4 months [95% CI 19.0–NA] vs. 16.1 months [95% CI 14.8–23.3] before IPTW, p=0.032). After IPTW adjustment, the RFS benefit remained significant (22.4 months [95% CI 18.8–NA] vs. 17.4 months [95% CI 14.6–20.6], p=0.036). Consistent results were observed in the sensitivity analysis using sIPTW (22.4 months [95% CI, 19.0–NA] vs. 17.4 months [95% CI, 14.6–20.6]; p=0.035). The 12-month RFS rates were 77.7% vs. 65.5% before IPTW, and 78.2% vs. 64.4% after IPTW. The 24-month RFS rates were 48.6% vs. 35.4% before IPTW, and 44.4% vs. 33.2% after IPTW (**Figure 2**, **Supplementary Figure S2A**). PSM generated 48 matched pairs, yielding in a matched cohort of 96 patients, with 48 patients in each group. Sensitivity analysis using PSM yielded consistent findings, with median RFS of 22.4 months (95% CI 18.8–NA) in the treatment group versus 17.5 months (95% CI 14.6–24.0) in the control group (p=0.039), confirming the robustness of the primary endpoint (**Supplementary Figure S3**). For OS, no statistically significant difference was observed between the two groups. The 24-month OS rates were 94.0% vs. 86.3% before IPTW and 93.6% vs. 83.4% after IPTW (p=0.150). Consistent results were observed after sIPTW (p=0.156) (**Figure 3**, **Supplementary Figure S2B**). The Schoenfeld residual tests indicated no significant violation of the proportional hazards assumption.

**Figure 2:**
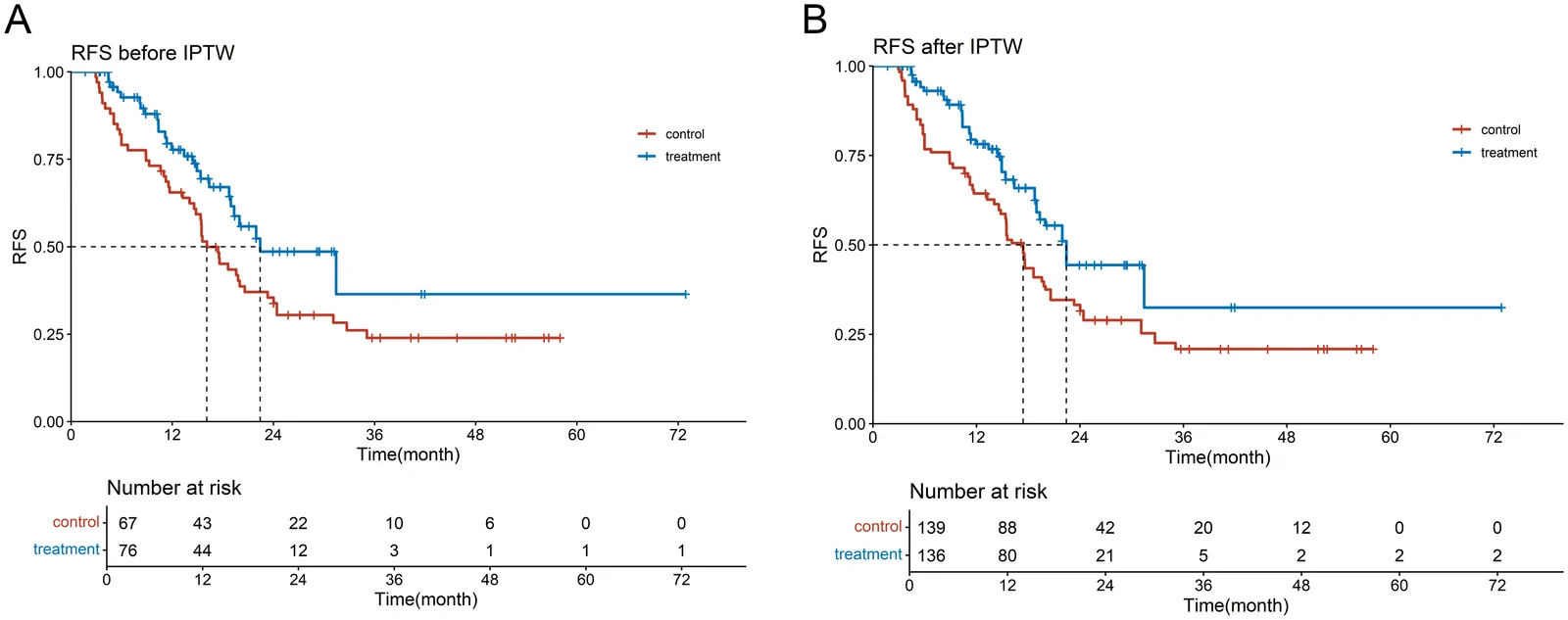
Kaplan–Meier curves for recurrence-free survival before and after propensity weighting **(A)** Unweighted cohort; **(B)** inverse-probability-of-treatment–weighted (IPTW) pseudo-population. Numbers at risk are shown below each plot. RFS, recurrence-free survival; IPTW, inverse-probability-of-treatment weighting.

**Figure 3:**
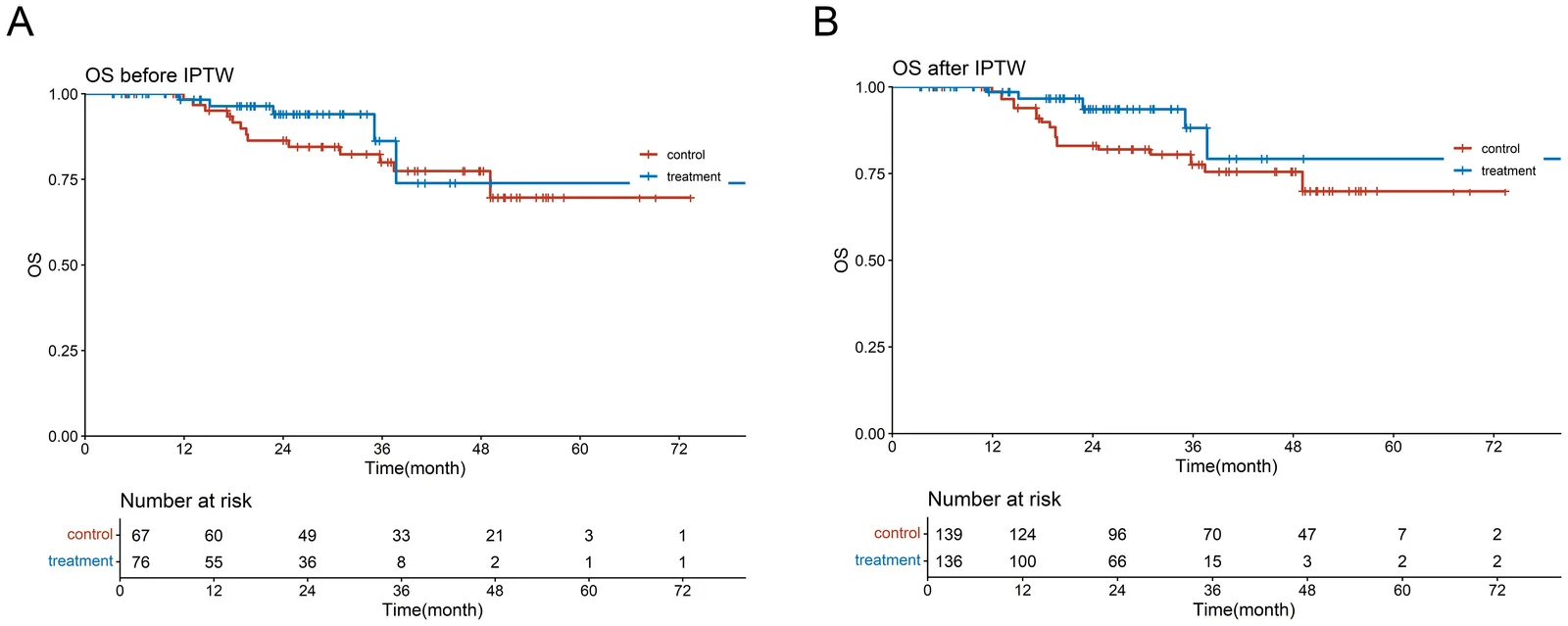
Kaplan–Meier curves for overall-survival before and after propensity weighting **(A)** Unweighted cohort; **(B)** inverse-probability-of-treatment–weighted (IPTW) pseudo-population; Numbers at risk are shown below each plot. OS, overall survival; IPTW, inverse-probability-of-treatment weighting; sIPTW, stabilized IPTW.

To further evaluate the potential influence of immortal time bias, a landmark analysis was conducted using a predefined time point of 8 weeks after surgery. Patients who remained recurrence-free at this landmark were included in the analysis. The IPTW-weighted Cox regression analysis showed that adjuvant therapy remained significantly associated with a reduced risk of recurrence (HR = 0.59, 95% CI 0.36–0.96, p = 0.03) (**Supplementary Figure S4**), suggesting that the primary findings were consistent after adjustment for potential time-related bias.

### Prognostic factors for RFS

Univariable analyses revealed that adjuvant donafenib plus sintilimab (HR = 0.59, 95% CI 0.37–0.96, p = 0.034) was significantly associated with longer RFS, while tumor diameter ≥5 cm showed a borderline adverse effect (HR = 1.66, 95% CI 0.99–2.80, p = 0.057). Other clinical variables, including age, sex, ECOG status, AFP ≥400 ng/mL, HBsAg positivity, PVTT, MVI, and tumor number, were not significantly associated with RFS (all p > 0.1). In the multivariable model before IPTW adjustment, adjuvant therapy remained independently associated with reduced recurrence risk (HR = 0.55, 95% CI 0.34–0.89, p = 0.016), whereas tumor diameter ≥5 cm emerged as a significant adverse prognostic factor (HR = 1.81, 95% CI 1.07–3.06, p = 0.027).

After IPTW weighting, these associations persisted. Univariable analysis again showed a significant association between adjuvant therapy and reduced recurrence risk (HR = 0.60, 95% CI 0.37–0.97, p = 0.037) and a borderline adverse effect of tumor diameter ≥5 cm (HR = 1.60, 95% CI 1.00–2.55, p = 0.050). In the IPTW-adjusted multivariable analysis, adjuvant therapy was independently associated with reduced recurrence risk (HR = 0.58, 95% CI 0.35–0.96, p = 0.036), while tumor diameter ≥5 cm remained independently associated with poorer RFS (HR = 1.65, 95% CI 1.01–2.70, p = 0.044) (**Figure 4**). Overall, these findings indicate that adjuvant donafenib combined with sintilimab was independently associated with improved RFS, whereas tumor diameter ≥5 cm was independently associated with poorer RFS in this cohort.

**Figure 4:**
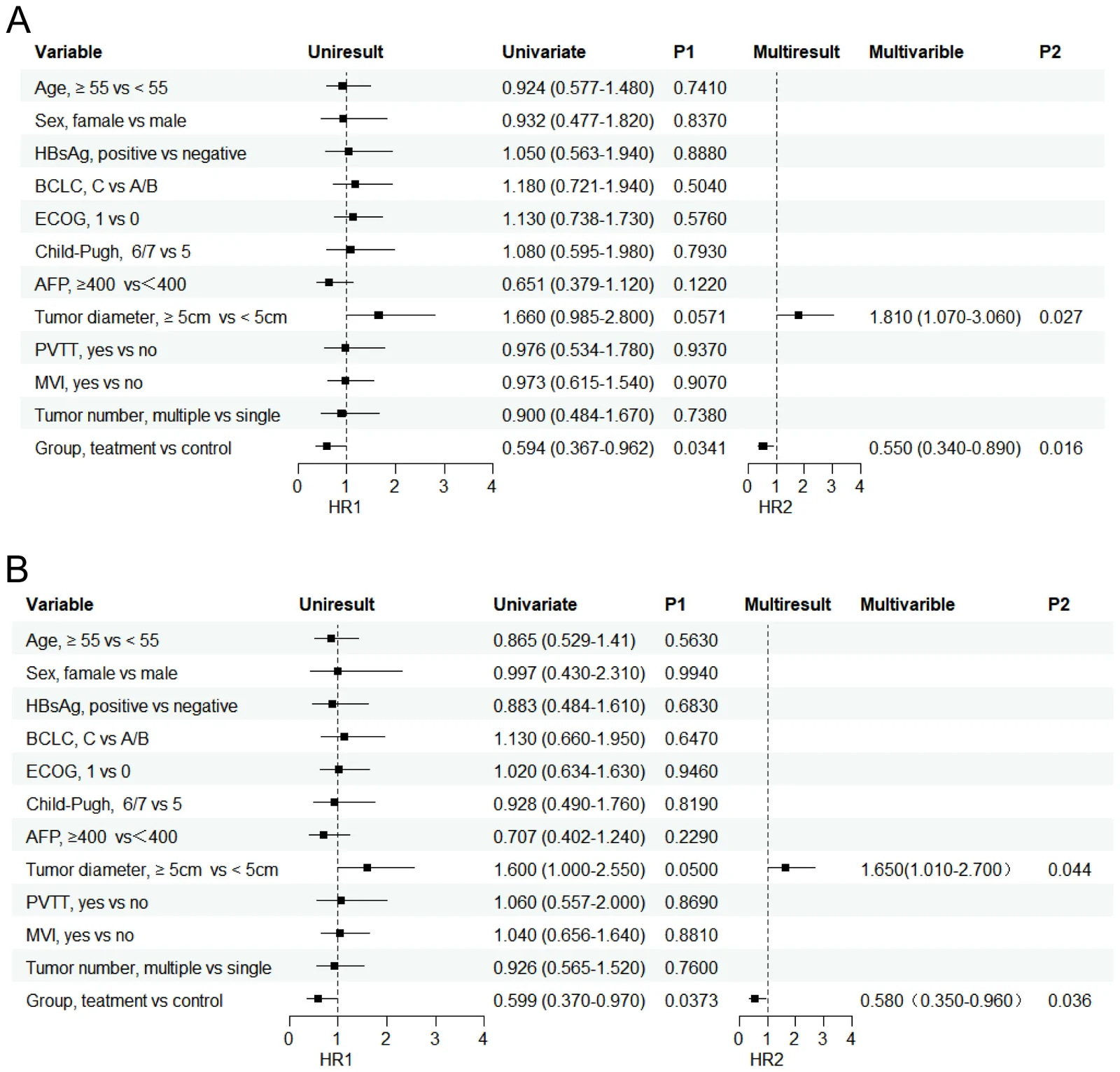
Univariable and multivariable analyses of recurrence-free survival (RFS) before and after IPTW **(A)** Univariable and multivariable analyses of RFS before IPTW; **(B)** Univariable and multivariable analyses of RFS after IPTW. Hazard ratios (HR) with 95% confidence intervals are shown. IPTW, inverse-probability-of-treatment weighting; RFS, recurrence-free survival.

### Subgroup analysis

Subgroup analyses were conducted to explore whether the treatment effect varied across patient subsets. After IPTW adjustment, a consistent trend of RFS benefit associated with donafenib plus sintilimab was observed in several key populations. Significant associations with a reduced recurrence risk were noted among male patients (p=0.027), those with AFP < 400 ng/mL (p=0.044), ECOG performance status of 0 (p=0.031), Child-Pugh score of 5 (p=0.029), and BCLC stage A/B disease (p=0.008) (**Figure 5**). Furthermore, as specifically requested to evaluate key clinicopathological features, postoperative adjuvant therapy was associated with a pronounced trend of RFS benefit in patients with a tumor diameter ≥ 5 cm (HR 0.47, 95% CI: 0.27–0.84, p=0.010), patients without MVI (HR 0.34, 95% CI: 0.15–0.74, p=0.007), and patients without PVTT (HR 0.45, 95% CI: 0.26–0.77, p=0.004). Regarding HBV status (HBsAg), although the 95% confidence intervals for both the positive and negative subgroups crossed 1.0 (positive: HR 0.85, 95% CI: 0.38–1.91; negative: HR 0.43, 95% CI: 0.14–1.32), the point estimates in both groups suggested a potential trend favoring adjuvant therapy. These findings indicate that the potential benefit of combined adjuvant therapy was not restricted to a single subgroup but extended across multiple clinically relevant strata. Importantly, no statistically significant interaction between treatment effect and subgroup variables was observed.

**Figure 5:**
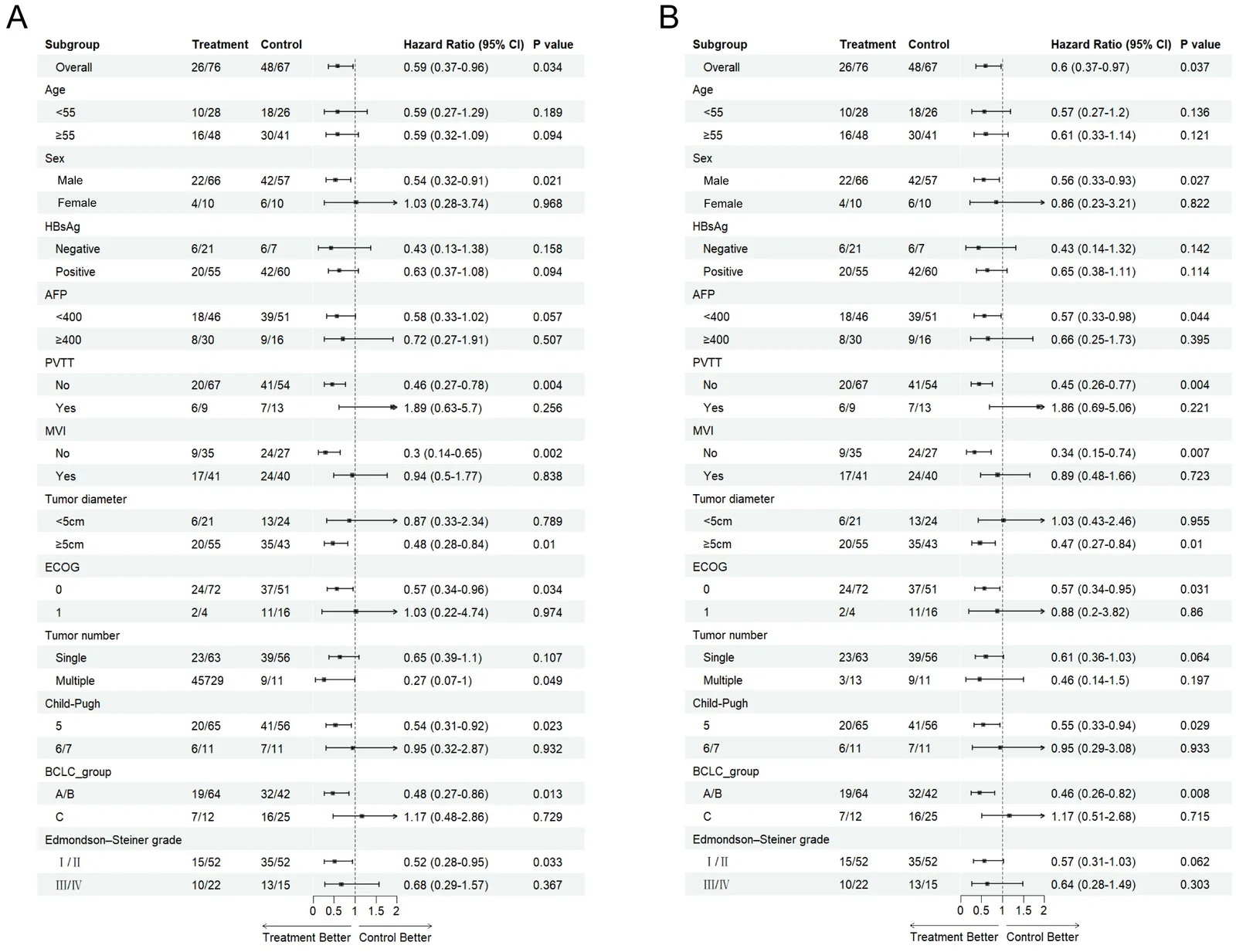
Forest plots of subgroup analyses of recurrence-free survival (RFS) before and after IPTW **(A)** Subgroup Cox regression analysis of RFS before IPTW; **(B)** Subgroup Cox regression analysis of RFS after IPTW. Hazard ratios (HR) with 95% confidence intervals are shown. RFS, recurrence-free survival; IPTW, inverse-probability-of-treatment weighting; HR, hazard ratio; CI, confidence interval; HBsAg, hepatitis B surface antigen; AFP, alpha-fetoprotein; PVTT, portal vein tumor thrombus; MVI, microvascular invasion; ECOG, Eastern Cooperative Oncology Group performance status; BCLC, Barcelona Clinic Liver Cancer.

### Safety

Safety analyses were performed in the 76 patients who received donafenib plus sintilimab. Overall, 72.4% of patients experienced at least one TEAE, and grade 3 events occurred in 7.9%. The most common adverse events were rash (31.6%), diarrhea (22.4%), hand-foot skin reaction (15.8%), and alopecia (13.2%). Less frequent events included fatigue (7.9%), thyroid dysfunction (6.6%), hypertension (5.3%), arthralgia (2.6%), oral mucositis (2.6%), decreased appetite (2.6%), and thrombocytopenia (2.6%). Single cases of AST elevation, colitis, and leukopenia were each reported (1.3%). Most adverse events were grade 1–2 and were managed with supportive care, treatment interruption, or dose adjustment. No treatment-related deaths were documented (**Table 2**).

**Table 2:** AEs in the donafenib combined with PD-1 adjuvant treatment group.

Frequency n (%)	Any grade TEAE	Grade 3 TEAE
Total	55(72.4)	6(7.9)
Rash	24(31.6)	1(1.3)
Diarrhea	17(22.4)	1(1.3)
Hand-foot skin reaction (HFSR)	12(15.8)	
Alopecia	10(13.2)	2(2.6)
Fatigue	6(7.9)	
Thyroid dysfunction	5(6.6)	1(1.3)
Hypertension	4(5.3)	
Arthrodynia	2(2.6)	1(1.3)
Oral mucositis	2(2.6)	
Decreased appetite	2(2.6)	
Thrombocytopenia	2(2.6)	
AST elevation	1(1.3)	
Colitis	1(1.3)	
Leukopenia	1(1.3)	

AE, adverse event; TEAE, treatment emergent adverse event.

The median documented duration of donafenib treatment was 11.8 months (IQR, 6.4–12.0 months), with duration data available for 74 of 76 patients. Overall, 33 patients (43.4%) received donafenib for at least 12 months. At the data cutoff, 11 patients remained on treatment but had not yet reached 12 months and were therefore considered not yet evaluable for treatment completion. Among the 63 evaluable patients, 33 (52.4%) completed at least 12 months of treatment. Donafenib dose reduction was documented in 7 patients (9.2%). Adverse event–related interruption or discontinuation of donafenib occurred in 21 patients (27.6%), comprising permanent discontinuation in 12 patients (15.8%) and temporary interruption followed by treatment resumption or continuation in 9 patients (11.8%). One additional patient temporarily interrupted treatment for approximately 2 weeks because of COVID-19 infection and subsequently resumed treatment.

Sintilimab interruption or discontinuation was documented in 17 patients; however, the underlying reasons were not consistently recorded and included non-toxicity-related factors. Consequently, adverse event–related permanent discontinuation of sintilimab and the incidence of individual immune-related adverse events could not be reliably quantified from the available retrospective records.

## Discussion

In this IPTW-balanced retrospective cohort study, adjuvant donafenib combined with sintilimab therapy was significantly associated with delayed postoperative recurrence in hepatocellular carcinoma patients carrying high-risk features, showed an early signal toward improved overall survival, and maintained a predominantly grade 1–2 safety profile. Collectively, these observations suggest that modern TKI–ICI therapy may represent a potential risk-adapted approach for addressing the unmet need for effective adjuvant options in resected HCC, and they provide a rationale for prospective trials to further evaluate and validate this strategy.

Because retrospective designs are prone to treatment-selection bias, we used IPTW and its stabilized form (sIPTW) to balance measured confounders; after weighting, covariate balance was substantially improved, with most baseline variables achieving or approaching a standardized mean difference of 0.10, mirroring the balance reported in contemporary IPTW applications for HCC outcomes research (16).

Hepatocellular carcinoma progression is closely linked to a complex tumor immune microenvironment characterized by immunosuppressive cell infiltration, chronic inflammatory signaling, and immune escape mechanisms that facilitate tumor growth and recurrence (17). Following liver resection, the host immune system may transiently shift toward an immunosuppressive state, accompanied by reduced immune surveillance and pro-inflammatory cytokine release, which may facilitate the outgrowth of residual microscopic disease and contribute to early postoperative recurrence (18). In HBV-related HCC, persistent viral antigen stimulation further promotes T-cell exhaustion and sustained activation of the PD-1/PD-L1 pathway, leading to immune tolerance within the tumor microenvironment and providing a biological rationale for PD-1 blockade in HBV-predominant populations (19). The use of anti-PD-1 monoclonal antibody in HBV-predominant populations may help restore antitumor immune responses by reinvigorating exhausted T cells.

Donafenib, a deuterated analog of sorafenib, inhibits multiple kinases including RAF, VEGFR-2, PDGFR-β and FLT3 while maintaining a favorable safety profile (20). Beyond its direct antitumor activity, inhibition of VEGF and Raf signaling pathways can remodel the tumor microenvironment. Anti-angiogenic therapy may promote vascular normalization, reduce the infiltration of immunosuppressive cell populations such as myeloid-derived suppressor cells (MDSCs) and regulatory T cells (Tregs), and alleviate tumor hypoxia, thereby converting immunologically “cold” tumors into a more immune-responsive or “hot” state (21). Parallel preclinical studies have also shown that sorafenib-class TKIs can enhance tumor antigen presentation and reduce immunosuppressive signaling, thereby increasing tumor susceptibility to immune checkpoint blockade (22, 23). This remodeled immune microenvironment provides a favorable context for PD-1 inhibitors to restore CD8^+^ T-cell function and promote durable antitumor immune responses. These complementary mechanisms provide a biological rationale for combining donafenib with an anti-PD-1 monoclonal antibody in the postoperative setting.

Surgical criteria in this study were based on the Chinese guidelines rather than those from Western countries (24). Notably, our cohort included selected patients with Vp1/2 portal vein tumor thrombus (PVTT) or hepatic vein tumor thrombus (HVTT) who would generally not have been considered surgical candidates under Western criteria but underwent curative-intent resection in accordance with Chinese guidelines (25). These patients represent a particularly high-risk postoperative population that remains underrepresented in many Western surgical and adjuvant studies, thereby extending the clinical relevance of our findings to a broader spectrum of resected HCC. Although the RFS benefit did not reach statistical significance in the PVTT subgroup, no significant treatment-by-PVTT interaction was observed, suggesting that the potential effect of adjuvant therapy should not be considered restricted by PVTT status. Accordingly, patients with vascular invasion remain an important population in whom intensified postoperative strategies warrant further prospective evaluation. Increasing evidence indicates that surgical resection can provide survival benefits for carefully selected patients with advanced HCC. Accordingly, several Asian guidelines, including those from China, recommend surgery as a first-line treatment option for selected patients with advanced disease (26, 27). After balancing covariates with IPTW, patients receiving donafenib + sintilimab in our cohort exhibited a longer median RFS compared with surveillance (median 22.4 vs 17.4 months), an association that remained in propensity-score–matched analyses and was accompanied by an emerging overall-survival (OS) separation. These results are broadly consistent with prior single-agent experiences. Wang et al. reported a median recurrence-free survival (RFS) of 27.7 months with adjuvant sintilimab versus 15.5 months with active surveillance in a randomized phase II trial of patients with resected hepatocellular carcinoma and microvascular invasion (28). In a separate single-arm study of adjuvant lenvatinib in patients with CNLC stage IIb/IIIa disease, the 12-month RFS rate was 60.4%, and the median RFS was 16.1 months (29), numerically lower than the values reported for sintilimab monotherapy and for our dual-agent regimen, which may suggest that TKI or ICI monotherapy alone is less effective in the highest-risk subsets, although this cross-study comparison is indirect and was not based on any formal statistical test. Collectively, these indirect cross-trial comparisons suggest that combined TKI–ICI strategies may have potential for reducing early recurrence in biologically aggressive hepatocellular carcinoma, and require confirmation in direct prospective comparisons.

The phase-III IMbrave050 trial provides a useful reference point: its early analysis showed a 28% RFS risk reduction with atezolizumab + bevacizumab (HR 0.72) (11), yet the 35-month update reported at ESMO 2024 revealed curve convergence (HR 0.90) and no OS gain, which may reflect a limited effect on late, *de-novo* relapse (12). In that trial, grade ≥3 adverse events occurred in ~41% of treated patients, including 2% treatment-related fatalities. In contrast, our combination produced mainly grade 1–2 events (hand–foot syndrome, alopecia), with no excess severe toxicities, consistent with the favorable hepatic safety profile previously reported for donafenib (13). However, given the differences in patient populations, treatment regimens, study designs, and follow-up duration, these cross-study comparisons are indirect and cannot establish the relative efficacy or safety of the two approaches. Taken together, these findings suggest that combining a TKI with an anti-PD-1 monoclonal antibody represents a biologically plausible adjuvant strategy that warrants further evaluation in prospective randomized trials, particularly among patients harboring multiple high-risk features.

Multivariable modeling identified two independent predictors of recurrence-free survival: receipt of adjuvant donafenib plus sintilimab and tumor diameter ≥ 5 cm. Importantly, subgroup analysis showed that the combination regimen was associated with the most marked reduction in relapse risk in patients with tumors ≥ 5 cm, a pattern also observed in other immunotherapy series. Chen et al. reported that adjuvant PD-1 monotherapy was significantly associated with improved RFS and OS in the ≥ 5 cm subset of high-risk patients (27), and postoperative lenvatinib plus PD-1 therapy was likewise associated with a lower incidence of early recurrence in the same size category (29). Together, these convergent data suggest that immune checkpoint inhibitor–based strategies may have potential relevance in patients with large, biologically aggressive tumors, thereby supporting further investigation of risk-adapted adjuvant approaches in hepatocellular carcinoma.

Notably, although differences in statistical significance were observed in subgroup analyses stratified by tumor size, MVI, and PVTT status (e.g., a significant association in the MVI-negative subgroup but a non-significant result in the MVI-positive subgroup), tests for interaction across these stratification factors were not statistically significant. This absence of significant interaction indicates that the treatment association does not exhibit statistical heterogeneity across these strata. The lack of statistical significance in certain subgroups (such as those with MVI or PVTT) is likely attributable to the limited sample sizes within these strata, resulting in reduced statistical power and wider confidence intervals. Therefore, the population potentially benefiting from this treatment should not be narrowly restricted to subgroups demonstrating statistical significance; rather, the potential benefit of this adjuvant strategy may extend to the broader high-risk population. Further prospective studies with larger sample sizes are warranted to validate these subgroup findings.

Several methodological limitations warrant consideration. The retrospective design introduces potential residual confounding, although a sizeable sample and median follow-up exceeding two years strengthen the dataset; IPTW was used to mitigate selection bias, but statistical adjustment cannot fully eliminate confounding from unmeasured variables, and minor residual imbalances may persist despite substantially improved covariate balance. The single-center design may also limit generalizability to populations with different viral etiologies or surgical practices. The exclusion of patients with recurrence or death within 3 months after surgery represents an additional potential source of selection bias: although intended to define a population that had adequately recovered and was clinically eligible for adjuvant therapy, this criterion may have preferentially excluded patients with occult residual disease, perioperative complications, or highly aggressive tumor biology—potentially underestimating true early recurrence and overestimating the apparent benefit of adjuvant therapy. Additionally, because treatment was initiated only after postoperative recovery, patients in the treatment group were required to remain recurrence-free before initiation, raising the possibility of immortal time bias; a landmark analysis from a prespecified time point yielded consistent results, although this bias cannot be entirely excluded. Safety assessment was further limited by the retrospective nature of data collection, as the reasons for sintilimab interruption or discontinuation and the occurrence of individual immune-related adverse events could not be reliably reconstructed, and the 12-month treatment completion rate should not be interpreted solely as a measure of tolerability, since duration may have been influenced by disease recurrence, patient preference, non-toxicity-related discontinuation, and the timing of the data cutoff. These caveats indicate that our findings should be interpreted as associations rather than causal effects, and prospective, multicenter randomized controlled trials are required to determine the clinical efficacy, optimal duration, sequencing, and dosing of TKI–ICI–based adjuvant therapy.

## Conclusions

Adjuvant donafenib plus sintilimab was associated with a reduced risk of early recurrence among high-risk HCC patients, with most documented AEs being grade 1–2 and no treatment-related deaths observed, suggesting that it represents a promising risk-adapted adjuvant strategy that merits priority evaluation in future prospective randomized trials.

## Data Availability

The original contributions presented in the study are included in the article/**Supplementary Material**. Further inquiries can be directed to the corresponding author.

## Supplementary material

Supplementary Figure 1 Distribution of propensity scores and covariate balance before and after adjustment. **(A)** Distribution of inverse probability of treatment weights (IPTW) with truncation limits indicated by red dashed lines. **(B)** Density distribution of propensity scores between the adjuvant donafenib plus sintilimab group and surveillance group before weighting. **(C)** Absolute standardized mean differences (SMDs) of baseline covariates before adjustment and after IPTW, stabilized IPTW (sIPTW), and propensity score matching (PSM). An SMD <0.1 was considered indicative of adequate covariate balance. IPTW, inverse probability of treatment weighting; sIPTW, stabilized inverse probability of treatment weighting; PSM, propensity score matching; SMD, standardized mean difference.

Supplementary Figure 2 Kaplan–Meier curves for survival outcomes after stabilized inverse probability of treatment weighting (sIPTW). **(A)** Stabilized IPTW-adjusted recurrence-free survival (RFS) curves comparing patients receiving adjuvant donafenib plus sintilimab therapy with those undergoing active surveillance. **(B)** Stabilized IPTW-adjusted overall survival (OS) curves between the two groups. Numbers at risk are shown below each Kaplan–Meier curve. sIPTW, stabilized inverse probability of treatment weighting; RFS, recurrence-free survival; OS, overall survival.

Supplementary Figure 3 Kaplan–Meier curves for recurrence-free survival before and after propensity score matching (PSM). **(A)** Recurrence-free survival (RFS) curves in the unadjusted cohort before PSM comparing patients receiving adjuvant donafenib plus sintilimab therapy with those undergoing active surveillance. **(B)** RFS curves in the PSM cohort after 1:1 nearest-neighbor matching between the two groups. Numbers at risk are shown below each Kaplan–Meier curve. PSM, propensity score matching; RFS, recurrence-free survival.

Supplementary Figure 4 Recurrence-free survival after an 8-week postoperative landmark. Kaplan–Meier curves compare patients who had initiated adjuvant donafenib plus sintilimab by day 56 after surgery (n = 71) with those who had not initiated treatment by day 56 (n = 66). Follow-up time is measured from the landmark. Tick marks indicate censored observations, and numbers at risk are shown below the plot. RFS, recurrence-free survival.
